# Case report on the marathon preparation of two middle-aged women aiming for a 4-hour finish

**DOI:** 10.3389/fphys.2025.1708925

**Published:** 2026-01-02

**Authors:** Bartosz Zając, Anna Mika, Anna Weronika Szablewska

**Affiliations:** 1 Laboratory of Functional Diagnostics, Central Scientific and Research Laboratory, University of Physical Culture in Krakow, Kraków, Poland; 2 Institute of Clinical Rehabilitation, University of Physical Culture in Kraków, Kraków, Poland; 3 Department of Obstetric and Gynecological Nursing, Institute of Nursing and Midwifery, Faculty of Health Sciences with the Institute of Maritime and Tropical Medicine, Medical University of Gdańsk, Gdańsk, Poland

**Keywords:** running, endurance training, physical endurance, exercise test, body composition, nutritional status, women

## Abstract

**Background:**

Recreational marathoners targeting the 4-h benchmark are underrepresented in the literature compared with elite runners, despite constituting a substantial share of participants. This case report documents the multifactorial, real-world preparation undertaken by two eumenorrheic middle-aged women and examines how training load, nutritional education, changes in body mass and composition, race-day weather conditions, and pacing strategy collectively contributed to an ∼20% improvement in performance.

**Methods:**

The retrospective analysis included training records from the 26 weeks preceding the marathon, outcomes of the Field-Based Running Test (lactate threshold velocity, mean velocity during the 5-min All-Out Trial, and maximal velocity), changes in body mass and composition, nutritional recommendations, race-day weather conditions, pacing strategy, and self-reported gastrointestinal symptoms.

**Results:**

Weekly running volume ranged 46–65 km, with a predominant frequency of four sessions per week. Both women completed two long runs ≥30 km during the final 4 weeks prior to the race. When classified relative to target marathon velocity, their intensity distributions appeared similar; however, the Field-Test–anchored system revealed clear differences: Woman A accumulated substantially more work in Zones 3–6, whereas Woman B trained proportionally more in Zones 1–2. Both athletes reduced body mass primarily through fat loss, but Woman A registered nearly twice the percentage decrease in absolute body mass and fat mass, and additionally showed a marked decline in muscle mass, accounting for roughly one-third of the total reduction. Both athletes improved their marathon performance by approximately 20%. Positive split pacing strategy observed in both cases. Neither athlete reported gastrointestinal problems during the marathon, held in 0.0 °C–2.2 °C air temperatures with wind speeds of 1.6–7.1 m s^–1^.

**Conclusion:**

This study demonstrates that a combination of prescribed training load, nutritional education, body mass reduction primarily through fat mass loss, as well as optimized pacing strategy can substantially improve marathon performance in middle-age women targeting a ∼4-h finish. Despite experiencing health issues in the final weeks of preparation, both participants achieved meaningful progress, underscoring the practical value of an integrated approach. Although not generalizable, these insights may assist coaches and athletes in designing effective preparation strategies for similar goals.

## Introduction

The marathon is among the most popular mass-participation disciplines, with about 1.1 million finishers recorded worldwide in 2018 (Andersen, 2024). Waśkiewicz et al. (2019) reported that personal goal achievement—such as improving running speed, competing with oneself, pushing personal limits, beating a specific time, or simply trying to run faster—emerged as the strongest motivation among these finishers. From a physiological perspective, performance is determined primarily by maximal oxygen uptake (V̇O_2_max), running efficiency, the ability to sustain a high fraction of V̇O_2_max over the duration of the effort (Joyner and Coyle, 2008), as well as physiological resilience (Jones and Kirby, 2025). These factors are, to some extent, modifiable through training (Bangsbo et al., 2025). It should be noted, however, that fully realizing an athlete’s potential also requires appropriate race management, including: (i) supplying energy to the working muscles (Barclay, 2017), (ii) replenishing water and electrolytes lost through sweat (Sawka et al., 2015), and (iii) adjusting pacing to the course profile and prevailing environmental conditions (Mantzios et al., 2022). In addition to these physiological and tactical determinants, sex-related differences warrant consideration, as they may significantly affect both performance outcomes and training responses in marathon runners.

Compared to men, women’s muscles typically generate 30%–50% less force and power, and their V̇O_2_max is on average 10%–30% lower, which results in lower outcomes in athletic performance when age and training status are matched (Hunter et al., 2023). Research further indicates that women are often less susceptible to muscle fatigue than men, particularly during isometric and slow dynamic contractions (Hunter, 2016), and some studies suggest faster recovery between sets of fatiguing exercise (Senefeld et al., 2018). Sex differences are also evident in aerobic adaptations: men generally achieve greater gains in both absolute and normalized V̇O_2_max compared to women (Howden et al., 2015).

Despite these differences, there are also notable similarities. Both sexes demonstrate comparable relative muscle hypertrophy and improvements in strength and power in response to high-load resistance training, observed in young adults (Roberts et al., 2020) as well as individuals over 50 years of age (Jones et al., 2021). Similarly, following sprint interval cycling, men and women exhibit similar relative increases in muscle mass and fatigue resistance (Bagley et al., 2021). This indicates that, although absolute performance levels differ, training principles can largely be applied in parallel, provided that sex-specific physiological characteristics are taken into account.

From a practical training perspective, it is necessary to consider the menstrual cycle in eumenorrheic women, as fluctuations in estrogen and progesterone concentrations may potentially influence performance and training responses. While hormonal changes may modulate metabolism and thermoregulation, their average impact on exercise capacity is relatively small (McNulty et al., 2020). For some women, however, dysmenorrhea or premenstrual symptoms may substantially limit exercise tolerance and performance (Taim et al., 2023). Individual responses throughout the cycle can further affect mood, pain perception, motivation, injury risk, and recovery, though these effects are highly variable (Taim et al., 2023). Training programs should therefore be adjusted to individual symptom profiles. In some athletes, menstrual disturbances such as secondary amenorrhea or oligomenorrhea may occur, often associated with relative energy deficiency in sport (Taim et al., 2023). This condition, considered a central component of the female athlete triad, arises from high training loads combined with insufficient energy intake and leads to reproductive dysfunction as well as other health consequences that may impair performance (Mathisen et al., 2023). Accordingly, preventive measures and medical consultation are strongly recommended, particularly for athletes engaged in intensive training (Mountjoy et al., 2023).

It is worth noting that, based on the current state of knowledge, the authors of this study state that the literature on marathon preparation has been disproportionately focused on elite runners, most often male. Recreational athletes—including women—who represent a substantial share of participants, remain underrepresented. At the 22nd TAURON Cracovia Marathon, approximately 31% of all finishers completed the race within 3:45–4:15 h. In this time range, women aged ≤59 years accounted for approximately 5% of all finishers (authors’ analysis of official results (Zieliński, 2025)). This illustrates the common clustering of performances around the 4-h benchmark. Assuming a similar distribution worldwide translates to approximately 340,000 runners per year achieving results within this range, including about 55,000 women aged ≤59 years—roughly equivalent to the capacity of a major football stadium. These figures highlight the need for more research on preparation strategies within this performance cluster.

Importantly, marathon preparation is multidimensional and subject to numerous potential confounders, which in real-world settings often makes it difficult to employ high-level-of-evidence study designs (e.g., cohort studies or randomized controlled trials). Consequently, researchers turn to case reports and case series, which not only maintain high ecological validity but also serve as a bridge connecting scientific research with practical application (Halperin, 2018). In line with this approach, the present article provides a comprehensive, longitudinal account of preparation strategies that yielded an approximately 20% improvement in marathon performance in two eumenorrheic middle-aged women aiming for a finish time around 4 h.

## Materials and methods

### Study design

This case report analyzed (i) training records from the 26 weeks preceding the marathon, (ii) anthropometric data, (iii) nutritional recommendations, (iv) outcomes of a Field-Based Running Test, (v) race-day weather conditions, and (vi) pacing strategies for two middle-aged women who prepared for the TAURON 22nd Cracovia Marathon, held on April 6, 2025. The data were compiled by a coach certified as an athletics instructor by the Polish Athletics Association, with research experience supported by numerous publications in peer-reviewed scientific journals. The around 4-h marathon goal (target marathon velocity (V_TM_) ∼2.93 m s^–1^) was proposed by the coach—based on previous race performances, anthropometric characteristics, the outcomes of the Field-Based Running Test, and the participants’ expectations—using heuristic decision making (Gigerenzer and Gaissmaier, 2011). During preparatory period participants completed the vast majority of sessions together. Additionally, the participants were asked to perform as many training sessions as possible in the shoes they intended to wear during the marathon, in order to ensure optimal adaptation and minimize the risk of discomfort on race day.

### Participants

The study involved the analysis of data from two eumenorrheic women. In both participants, complete blood count parameters and indicators of iron status (total iron-binding capacity, serum iron concentration, and ferritin (Kuwabara et al., 2022)) were within reference ranges. The athletic level of the participants can be classified as Trained/Developmental according to McKay’s classification (McKay et al., 2022). This classification is supported by indicators such as skill development, training with the purpose of competition, identification with a specific sport, regular training approximately three times per week, and representation at the local level. Both participants combined marathon training with intellectually demanding professional work. Prior to commencing the marathon preparation process, the participants submitted a medical statement confirming the absence of contraindications to endurance training, competitive participation, and exercise testing. The baseline basic characteristics of the participants are presented in Table 1.

**TABLE 1:** Baseline basic characteristics.

Variable	Woman A	Woman B
Age [years]	40	50
Body height [m]	1.69	1.58
Body mass (BM) [kg]	72.1	66.0
Fat-free mass [kg (%BM)]	51.0 (70.7)	45.3 (68.6)
Fat mass [kg (%BM)]	21.1 (29.3)	20.7 (31.4)
Training experience [years]	5	5
Personal best [h:mm:ss (m·s^-1^) [%V_TM_]]		
5k	0:24:37 (3.39) [115.5]	0:23:56 (3.48) [118.8]
10k	0:52:27 (3.18) [108.4]	0:52:31 (3.17) [108.3]
Half marathon	1:56:00 (3.03) [103.4]	1:55:16 (2.97) [104.1]
Marathon	4:42:51 (2.49) [84.8]	4:50:35 (2.43) [82.6]
Lactate threshold velocity [m·s^-1^ (%V_TM_)]^a^	2.44 (83.3)	3.12 (106.5)
5-min all-out trial [m·s^-1^ (%V_TM_)]^a^	3.32 (113.3)	3.46 (118.1)
Maximal velocity [m·s^-1^ (%V_TM_)]^a^	6.60 (225.2)	5.87 (200.3)

^a^ Lactate threshold velocity, mean velocity from the 5-min All-Out Trial, and maximal velocity were assessed during the Field-Based Running Test, as described in the “Field-Based Running Test” section. Abbreviation: V_TM_—target marathon velocity.

### Anthropometric measurements

Anthropometric measurements were performed each time in sportswear and barefoot, before training, at approximately 5:00 p.m., after the consumption of two meals (breakfast and lunch) and following bladder and bowel voiding immediately prior to measurement. Participants were instructed to maintain consistent dietary habits on the days of scheduled assessments, in accordance with the pattern established during the first measurement. The described measurement conditions were determined by logistical constraints. Body height was measured using a stadiometer (Seca 213, Seca GmbH & Co. KG, Germany) with an accuracy of 1 cm. Body mass (BM) and body composition were assessed using a bioelectrical impedance analyzer (Tanita DC-430MA, Tanita Corp., Japan) with an accuracy of 0.1 kg, in accordance with the manufacturer’s guidelines.

### Field-based running test

**FIGURE 1:**
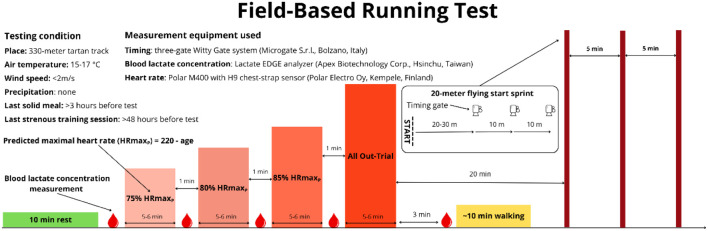
Field-based running test protocol.

The field-based running test was used to establish the physiological profile of the participants, specifically to determine lactate threshold velocity (V_LT_), mean velocity during the 5-min All-Out Trial (V_AOT_)—corresponding to maximal aerobic velocity (Berthon et al., 1997), and maximal velocity (V_MAX_). The detailed protocol is illustrated in Figure 1. The test began with 10 min of passive rest. Participants then performed four progressive intervals lasting between 5 and 6 min of running, after which they were asked to report their perceived exertion using the Borg 6–20 scale (Williams, 2017). Split times for successive 165-m segments within each interval were recorded. For the first three intervals, the mean running velocity used for analysis was calculated from the last two complete segments, whereas in the fourth interval the mean velocity across the entire bout was analyzed. Therefore, a 5–6 min time window was applied to ensure that each interval could be concluded only upon completion of the last full 165-m segment. For the first three intervals, exercise intensity was self-regulated by the participants using continuous heart rate monitoring with a Polar M400 watch and an H9 chest-strap sensor (Polar Electro Oy, Kempele, Finland). Therefore, they were instructed not to start too fast and to keep their heart rate as close as possible to the target value, being reminded of the kinetics of the heart rate response (Bunc et al., 1988). Exceeding the lactate threshold was defined as an increase in blood lactate concentration of at least 1 mmol·L^−1^ compared to the first interval (Hagberg and Coyle, 1983). In both cases, the threshold was exceeded during the third interval (85% predicted HR_MAX_); however, in the event of non-exceedance, an additional stage at 90% predicted HR_MAX_ was scheduled. The fourth interval consisted of an All-Out Trial, during which participants were instructed to cover the greatest possible distance. The trial was considered valid if peak heart rate exceeded 90% of predicted HR_MAX_, post-exercise blood lactate concentration was greater than 8 mmol·L^−1^, and the Borg scale rating was above 18 (Midgley et al., 2007). Twenty minutes of passive-active recovery (Ortiz et al., 2019) after the completion of the All-Out Trial, maximal sprinting velocity was assessed using a 20-m flying-start sprint, during which the times of two consecutive 10-m segments were recorded. The obtained values may have been underestimated due to residual fatigue; however, logistical considerations determined this protocol. The initial approach run was set at 25 m. Mean velocities were calculated for each segment and compared; if the mean velocity in the second segment exceeded that of the first by ≥ 0.05 m·s^−1^, the approach run was extended to 30 m, whereas if the mean velocity in the first segment was lower than that of the second by ≥ 0.05 m·s^−1^, the approach run was shortened to 20 m. This procedure allowed for the adjustment of the approach run to the individual acceleration characteristics of each participant (Korhonen et al., 2003). Participants were instructed to accelerate as quickly as possible and maintain maximal velocity across three timing gates. The fastest 10-m split was used for analysis.

### Training load recording

**FIGURE 2:**
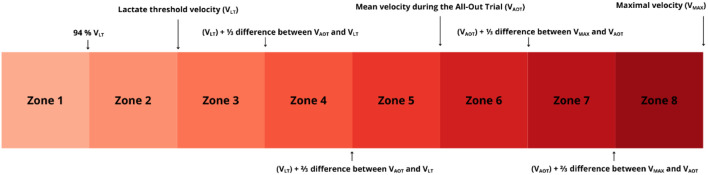
Intensity classification based on the field-based running test.

**FIGURE 3:**
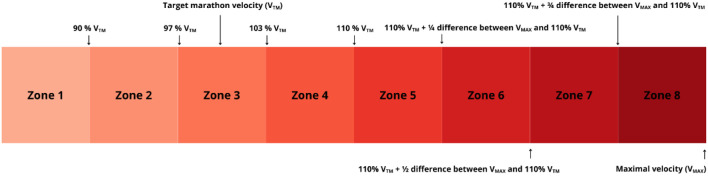
Intensity classification based on target marathon pace.

The training load analysis was based on data including records from Garmin Forerunner 965 sports watches (Garmin Ltd., Olathe, KS, United States) (Szot et al., 2021), and additional information collected during supervised training sessions and through informal communication with the participants. All data were gathered by the coach in charge. Training volume was expressed in kilometers, while intensity was classified using two complementary systems of eight zones (Z1–Z8). The first system was anchored to variables obtained from the Field-Based Running Test, while the second was anchored to the V_TM_. Detailed information on the adopted classification systems is presented in Figures 2, 3. To allocate completed kilometers to the appropriate intensity zones, two complementary approaches were employed depending on the type of training session. In the case of continuous runs, the number of kilometers performed in each intensity zone was determined based on the average running speed recorded by the participants’ sports watches equipped with GPS. The average velocity calculated for the entire session or its segments was used to assign each portion of the run to a specific intensity zone. In contrast, for interval-based sessions performed on pre-measured segments, intensity classification was based on the average velocity of each repetition, calculated from the time taken to cover the given distance. This dual approach ensured both accuracy and alignment with real-world training conditions. For analytical consistency, warm-ups (including 2 km of running and 1 km of walking drills) were standardized as 3 km in Z1, and cool-downs (2 km of running at Zone 1 intensity) were coded as 2 km in Z1. As the training program consisted exclusively of running sessions, no analysis of training load by activity type was performed.

### Nutritional guidelines

To support attainment of the intended performance goal, participants were advised to reduce body mass (BM) by ∼4–5% from baseline over a 26-week period—primarily by reducing fat mass (FM) — given the well-documented sensitivity of long-distance running performance to body mass (Dennis and Noakes, 1999). The target body-fat percentage for both participants remained above the ACSM-recommended minimum safe level of 14% for women (Ozemek et al., 2025). Specifically, assuming that 1 kg of fat mass corresponds to ∼7,000 kcal (Finkler et al., 2012), a reduction of 2.9–3.6 kg in Woman A required an energy deficit of ∼20,300–25,200 kcal (i.e., 112–138 kcal·day^−1^), while a reduction of 2.6–3.3 kg in Woman B corresponded to ∼18,200–23,100 kcal (i.e., 100–124 kcal·day^−1^). Participants were instructed that energy balance could be monitored using the estimated daily energy expenditure provided by the sports watches (Kinnunen et al., 2019) and self-reported daily energy intake (EI) recorded with commercial smartphone application (Ambrosini et al., 2018). They also received dietary guidelines on macronutrient distribution (Thomas et al., 2016). Recommended EI from protein was 15%–25% of total EI (1.6–2.2 g · kg BM^−1^ day^−1^), from fat 20%–35% of EI (approximately 1–1.5 g kg BM^−1^ day^−1^), and from carbohydrates 50%–60% of EI (approximately 5–7 g kg BM^−1^ day^−1^). Although EI was not directly monitored during the study, BM and body composition were assessed at 4-week intervals. On weekends, when the longest training sessions were scheduled, participants were asked to consume breakfast approximately 3 h before the start of training, which was conducted at the same time of day as the planned marathon start. They were instructed to choose familiar and well-tolerated carbohydrate-rich foods that they personally preferred, in order to minimize gastrointestinal discomfort (Martinez et al., 2023) and ensure adequate energy availability (Thomas et al., 2016). The composition of these pre-training meals was aligned with the breakfast planned for race day. In the final 48 h before the marathon, participants were advised to follow a carbohydrate-loading protocol, aiming for a carbohydrate intake of 10–12 g kg BM^−1^ · day^−1^ (Thomas et al., 2016). During this period, recommendations included avoiding hard-to-digest foods and paying particular attention to hydration. Pre-race hydration was recommended as approximately 5–10 mL kg BM ^−1^ of fluids consumed between four and 2 h before the start (Thomas et al., 2016). To minimize the likelihood of gastrointestinal problems, participants were advised to fully empty both bowel and bladder 20–30 min before the start. During the race, participants were advised to consume approximately 100–150 mL of fluids every 5 km (adjusted to air temperature on race day) and to ingest one energy gel containing 30 g of carbohydrates (glucose:fructose ratio 1-2:1 (Rowlands et al., 2015)) every 7 km, starting from the 10th km (Thomas et al., 2016). During the marathon preparation period, participants were recommended vitamin D_3_ supplementation, administered in doses specified by the manufacturers. This supplementation did not require medical supervision or monitoring (Thomas et al., 2016). It was particularly important, as the training period coincided with the autumn–winter months in a Polish city located at approximately 50 °N, where sunlight exposure is limited (Rusińska et al., 2018). During key training sessions identified by the coach, athletes were encouraged to practice race-day fueling strategies, including the intake of energy gels and fluids. This was intended not only to prepare the gastrointestinal system for digesting these products during physical exertion (Martinez et al., 2023), but also to rehearse the technical execution of consuming gels and fluids efficiently under race conditions.

### Race analysis

The analysis of running velocity was based on official and publicly available split-time data obtained through electronic timing systems employed by the marathon organizer (Zieliński, 2025). The primary course profile parameters (minimum, average, and maximum altitude) were obtained from the official race organizer’s website, and these data were supplemented with a measurement of the total elevation gain recorded using a participant’s sports watch (Szot et al., 2021). Meteorological data were obtained from the AGH University of Krakow (Herman, 2018). As the measurements originated from a source not certified by the World Meteorological Organization, minor uncertainties should be considered when interpreting the results. After the race, both women were also asked to report whether they had experienced any gastrointestinal problems during the marathon.

## Results

### Body mass and composition

**FIGURE 4:**
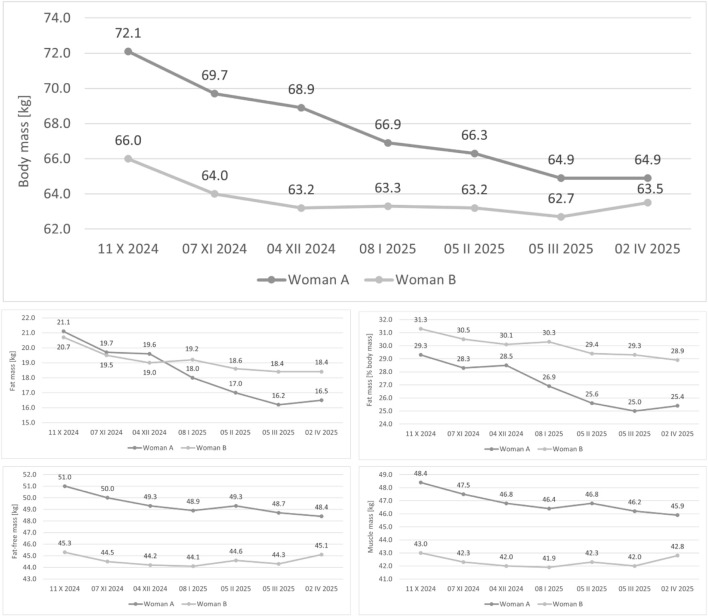
Time-course of body mass and body composition indices across the 26-week training program.

Over the 26-week period, BM [kg] in Woman A decreased by 7.2 kg (−10.0%), fat mass [% BM] decreased by 3.9 percentage points (−13.3%), fat mass [kg] was reduced by 4.6 kg (−21.8%), fat-free mass [kg] decreased by 2.6 kg (−5.1%), and muscle mass [kg] declined by 2.5 kg (−5.2%). In Woman B, BM [kg] decreased by 2.5 kg (−3.8%), fat mass [% BM] decreased by 2.4 percentage points (−7.7%), fat mass [kg] was reduced by 2.3 kg (−11.1%), fat-free mass [kg] remained stable with a change of −0.2 kg (−0.4%), and muscle mass [kg] decreased by 0.2 kg (−0.5%). Detailed data on the values of individual parameters at each measurement point are presented in Figure 4.

### Training load analysis

**FIGURE 5:**
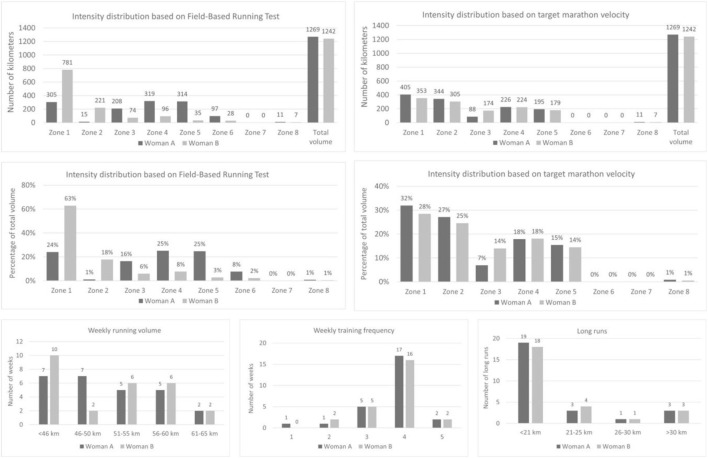
Characteristics of training load.

The analysis showed that, over the study period, both women completed training of comparable total volume and similar intensity distribution when classified according to target marathon velocity. However, applying an alternative approach—intensity classification based on the Field-Based Running Test—revealed clear differences between the participants. Woman B performed a relatively greater proportion of training in Zones 1–2, whereas Woman A accumulated substantially more training volume in Zones 3–6. With respect to weekly running volume, Woman A experienced fewer weeks with less than 46 km, while more frequently maintaining a volume within the 46–50 km range compared with Woman B. With respect to long runs, both athletes completed two sessions of at least 30 km during the final 4 weeks prior to the marathon race, excluding the race itself. In terms of training frequency, the predominant pattern for both athletes was four sessions per week. These patterns are illustrated in Figure 5.

The detailed training program is presented in Table 2. It is noteworthy that both participants experienced health issues between weeks 21 and 23. Woman A suffered from an upper respiratory tract infection, whereas Woman B sustained an overuse injury affecting the lower leg. In both cases, the diagnoses were provided by medical professionals with whom the athletes consulted during this period.

**TABLE 2:** Detailed training program description.

Week	Day	Training session description
1	1	CR 10 km (Z1)
	2	x
	3	Field-based running test
	4	x
	5	CR 6 km (Z3)^a^
	6	x
	7	CR 15 km (Z1)
2	1	CR 10 km (Z1)
	2	x
	3	IR 2 × 3 km (Z4) R: 3 min^a^
	4	x
	5	CR 6 km (Z1) + IR 6 × 100 m (Z8) R: 90s
	6	x
	7	Competition: half-marathon
3	1	CR 10 km (Z1)
	2	x
	3	IR 2 × 10 × 200 m (Z5) R: 45 s/3 min^a^
	4	x
	5	CR 7 km (Z4)^a^
	6	x
	7	CR 15 km (Z1)
4	1	CR 10 km (Z1)
	2	x
	3	IR 15 × 330 m (Z5) R: 50 s^a^
	4	x
	5	CR 8 km (Z3)^a^
	6	x
	7	CR 15 km (Z2)
5	1	CR 10 km (Z1)
	2	x
	3	CR 6 km (Z1) + IR 6 × 100 m (Z8) R: 90 s
	4	IR 3 × 2 km (Z5) R: 3 min^a^
	5	x
	6	CR 6 km (Z1) + IR 6 × 100 m (Z8) R: 90 s
	7	Competition: 10 km^a^
6	1	x
	2	CR 8 km (Z1) + IR 8 × 100 m (Z8) R: 90 s
	3	IR 12 × 400 m (Z5) R: 60 s^a^
	4	x
	5	CR 9 km (Z3)^a^
	6	x
	7	CR 15 km (Z3)
7	1	CR 10 km (Z1)
	2	x
	3	IR 7 × 660 m (Z5) R: 2 min^a^
	4	x
	5	CR 10 km (Z3)^a^
	6	x
	7	CR 15 km (Z1)
8	1	CR 10 km (Z1)
	2	x
	3	IR 5 × 1 km (Z5) R: 2 min^a^
	4	x
	5	CR 6 km (Z1) + IR 6 × 100 m (Z8) R: 90 s
	6	Competition: 5 km^a^
	7	x
9	1	CR 10 km (Z1)
	2	x
	3	IR 3 × 2 km (Z5) R: 3 min^a^
	4	CR 8 km
	5	CR 11 km (Z3)^a^
	6	x
	7	CR 15 km (Z1)
10	1	CR 10 km (Z1)
	2	x
	3	IR 4 × 2 km (Z5) R: 3 min^a^
	4	x
	5	CR 12 km (Z3)^a^
	6	x
	7	CR 15 km (Z2)
11	1	CR 10 km (Z1)
	2	x
	3	CR 15 km (Z3) [A]
	4	x
	5	IR (2 × 3 km) + 2 km (Z5) R: 3 min^a^
	6	x
	7	CR 13 km (Z4)^a^
12	1	x
	2	CR 6 km (Z1) + IR 6 × 100 m (Z8) R: 90 s
	3	IR 3 × 3 km (Z4) R: 4 min^a^
	4	x
	5	CR 15 km (Z2)
	6	x
	7	CR 14 km (Z4)^a^
13	1	x
	2	CR 10 km (Z1)
	3	x
	4	CR 6 km (Z1) + IR 6 × 100 m (Z8) R: 90 s
	5	IR 3 × 3 km (Z5) R: 4 min^a^
	6	x
	7	CR 15 km (Z3)
14	1	x
	2	CR 6 km (Z1) + IR 6 × 100 m (Z8) R: 90s
	3	IR 4 km+3 km+2 km+1 km (Z5) R: 3min^a^
	4	x
	5	CR 15 km (Z2)
	6	x
	7	CR 15 km (Z3)
15	1	x
	2	CR 6 km (Z1) + IR 6 × 100 m (Z8) R: 90s
	3	CR 15 km (Z2)
	4	x
	5	IR (2 × 4 km)+2 km (Z5) R: 4 min^a^
	6	x
	7	CR 16 km (Z4)
16	1	x
	2	CR 6 km (Z1) + IR 6 × 100 m (Z8) R: 90s
	3	IR 5 km+4 km +1 km (Z5) R: 5 min^a^
	4	x
	5	CR 15 km (Z2)
	6	x
	7	CR 17 km (Z4)
17	1	x
	2	CR 6 km (Z1) + IR 6 × 100 m (Z8) R: 90s
	3	IR 2 × 5 km (Z5) R: 5 min^a^
	4	x
	5	CR 12 km (Z4)
	6	x
	7	CR 25 km (Z2)
18	1	x
	2	CR 6 km (Z1) + IR 6 × 100 m (Z8) R: 90s
	3	IR (2 × 5 km) + 1 km (Z5) R: 5 min^a^
	4	x
	5	CR 15 km (Z2)
	6	x
	7	CR 18 km (Z4)
19	1	x
	2	CR 6 km (Z1) + IR 6 × 100 m (Z8) R: 90 s
	3	IR (2 × 5 km) + 2 km (Z5) R: 5 min^a^
	4	x
	5	CR 27 km (Z2)
	6	x
	7	x
20	1	CR 6 km (Z1) + IR 6 × 100 m (Z8) R: 90 s
	2	x
	3	IR 6 km+5 km+1 km (Z5) R: 4 min^a^
	4	x
	5	CR 15 km (Z2)
	6	x
	7	CR 19 km (Z4)
21	1	[A] [B]
	2	[A] [B]
	3	[A] [B]
	4	[A] [B]
	5	[A] [B]
	6	[A] [B]
	7	[A] [B]
22	1	[A] [B]
	2	CR 6 km (Z1) + IR 6 × 100 m (Z8) R: 90 s
	3	IR 3 km+1 km (Z5) R: 4 min^a^ [B]
	4	[A] [B]
	5	[A] [B]
	6	[A] [B]
	7	Competition: 10 km^a^
23	1	x
	2	x
	3	CR 10 km (Z1) [A]
	4	x
	5	CR 5 km (Z5)^a^
	6	x
	7	CR 30 km (Z2)
24	1	x
	2	CR 6 km (Z1) + IR 6 × 100 m (Z8) R: 90 s
	3	IR 3 × 2 km (Z5) R: 5 min^a^ [B]
	4	x
	5	x
	6	Competition: half-marathon
	7	x
25	1	x
	2	CR 6 km (Z1) + IR 6 × 100 m (Z8) R: 90 s
	3	CR 10 km (Z1)
	4	x
	5	IR 4 × 2 km (Z5) R: 5 min^a^ [B]
	6	x
	7	CR 32 km (Z2)
26	1	x
	2	CR 6 km (Z1) + IR 6 × 100 m (Z8) R: 90 s
	3	IR 3 × 2 km (Z5) R: 5 min^a^
	4	x
	5	x
	6	x
	7	Competition: 22nd cracovia marathon

Abbreviations: CR, continous run; IR, intermittent run, Z1-8 – intensity zone based on V_TM_, R–passive rest interval (between repetitions/sets).

^a^ training session preceded by a warm-up and followed by a cool-down, x–rest day, [A]/[B] – training session not completed by Woman A/B due to health issues.

### Race analysis

**FIGURE 6:**
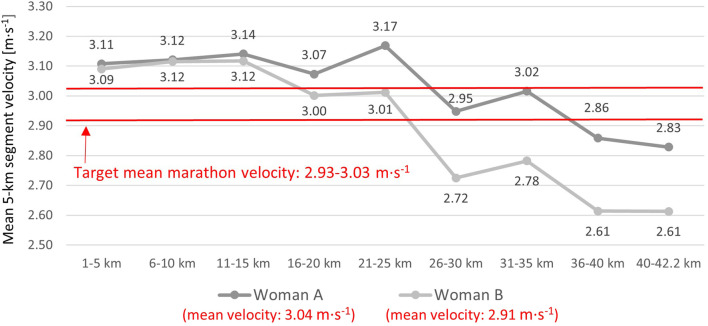
Pacing profile across the marathon race.

Participants were instructed to maintain an even running velocity within the target range (2.93–3.03 m·s^−1^). Woman A ran above the upper bound of this range until ∼25 km, whereas Woman B did so until ∼15 km. Thereafter, mean velocity fell below the pre-specified target zone after the 35th kilometer for Woman A and after the 25th kilometer for Woman B. Figure 6 illustrates these pacing dynamics. Relative to pre-race assessments, mean marathon velocity increased by 22.1% for Woman A and by 19.8% for Woman B. The total elevation gain during the race was 134 m, with a minimum altitude of 199 m, an average altitude of 206 m, and a maximum altitude of 220 m. Race-day conditions were as follows: air temperature ranged from 0.0 °C to 2.2 °C, wind speed from 1.6 to 7.1 m·s^−1^, and no precipitation was recorded. Neither of the women reported experiencing gastrointestinal problems during the race.

## Discussion

The present case study shows that a multifactorial intervention—comprising prescribed training load, nutritional education, regular monitoring of body mass and body composition, and the race-day pacing strategy adopted—was associated with substantial improvements in marathon performance. Specifically, Woman A achieved a 22.1% increase in mean marathon velocity, finishing in 3:51:27, while Woman B improved by 19.8% with a final time of 4:02:18 relative to their pre-race assessments. It is noteworthy that both participants experienced health-related issues between weeks 21 and 23 of the preparation period, indicating that the preparation process was not entirely free of complications. Nevertheless, both athletes achieved performances close to or below the 4-h benchmark. The integrated approach described here may therefore serve as a source of inspiration for recreational athletes and coaches aiming to prepare for performances around this widely pursued goal.

The substantial performance improvements observed in both participants can be attributed to several complementary elements of the intervention. First, the applied training load—comprising a weekly mileage typically ranging from 46 to 65 km, an average frequency of four training sessions per week, and two long runs of at least 30 km performed within the final 4 weeks before the marathon—likely provided a sufficient stimulus to prepare the body for completing the race in approximately 4 h. It should be emphasized, however, that Woman B sustained an overuse injury during the final stage of her preparation. This indicates that the cumulative training load—interacting with lifestyle factors and her individual morphological and biomechanical characteristics—exceeded the load tolerance of her musculoskeletal system (Correia et al., 2024). Preventing recurrence requires an accurate diagnosis and a clear understanding of the injury mechanism, which should then form the basis for a targeted intervention. Depending on the diagnosis, measures may include: (i) adjusting training-load parameter values (e.g., volume, intensity, or frequency), (ii) modification of lifestyle to reduce non-training stressors (e.g., reducing occupational workload) and to increase free time devoted to passive rest, sleep (Huang and Ihm, 2021) or incorporating daytime naps (Mesas et al., 2023), (iii) optimizing running technique, or (iv) implementing methods aimed at accelerating post-exercise recovery (Dupuy et al., 2018). Second, the nutritional recommendations, combined with regular monitoring of BM and body composition, likely contributed indirectly to BM reduction in both women, primarily through fat loss—a change that, within these ranges, can be considered beneficial in the context of marathon preparation (Mathisen et al., 2023). However, the marked decline in muscle mass (accounting for nearly one-third of the reduced body mass) observed in Woman A cannot be regarded as advantageous and may have resulted from a combination of low energy availability and insufficient protein intake—factors that could have potentially contributed to impaired immune function (Mountjoy et al., 2023) which in turn might have facilitated the onset of an upper respiratory tract infection. These observations highlight the need for more detailed monitoring of energy availability and macronutrient distribution during periods of high training load. Third, carbohydrate loading over the final 48 h may have maximized pre-race glycogen stores, complemented by in-race carbohydrate gels to sustain energy availability. In the cool conditions in which the race was held (0.0 °C–2.2 °C), hydration demands were low (Jenkins et al., 2023), although a minimal fluid intake was maintained to support metabolic and thermoregulatory processes. Notably, the absence of gastrointestinal issues during the race—which are commonly reported among endurance athletes (de Oliveira et al., 2014) —may be attributed to deliberate preparatory strategies. Both participants practiced the intake of carbohydrate gels and fluids during training, which likely helped their gastrointestinal systems adapt to fueling under exercise conditions (Martinez et al., 2023). The race-day breakfast had been pre-tested, and avoiding difficult-to-digest foods in the 48 h preceding the race likely provided further protection against exercise-associated gastrointestinal symptoms.

Several factors may explain why Woman A achieved a greater relative improvement in marathon performance compared with Woman B. First, Woman A experienced more than twice the percentage reduction in absolute BM and nearly twice the percentage decrease in absolute fat mass. In a subsequent marathon preparation cycle, Woman B could benefit from further body mass reduction—primarily through fat mass loss—since her pre-race level was well above the ACSM-defined minimal threshold of 14% body fat for women (Ozemek et al., 2025). Second, analysis of training load indicated that Woman A accumulated substantially more volume in Zones 3–6, whereas Woman B performed a greater proportion of training in lower-intensity Zones 1–2. In a subsequent preparation cycle, it might be beneficial for Woman B to maintain a similar overall training volume but increase the proportion of work performed in Zones 3–6, while keeping in mind the need to carefully manage injury risk. Third, age may also have influenced the preparation process. Woman B was 10 years older than Woman A, which could have contributed to slower recovery processes (Li et al., 2024), thereby indirectly reducing responsiveness to training load. These data again suggest the need for a revision of both training and non-training stressors in Woman B, as well as the implementation of interventions aimed at accelerating post-exercise recovery. In addition, given the age of Woman B, it is possible that she was approaching or undergoing the perimenopausal period (Appiah et al., 2021), which may be associated with hormonal fluctuations and, consequently, could contribute to challenges in recovery (Pellegrino et al., 2022) and body mass regulation (Chopra et al., 2019). Finally, in the initial phase of the race, both women maintained a velocity above the upper bound of the target velocity range. For Woman A, this aggressive opening proved beneficial—she finished with an average velocity exceeding that bound, suggesting the target range was underestimated for her under the race-day conditions. In contrast, Woman B’s fast start likely led to a more pronounced slowdown in the second half of marathon, resulting in an average velocity slightly below the lower bound of the target range, which may be considered a tactical pacing error in her case (Skorski and Abbiss, 2017).

### Strengths and limitations

This study has several notable strengths. It provides a comprehensive and multifactorial perspective on marathon preparation by integrating training load analysis, nutritional strategies, supplementation, body composition monitoring, and race strategy. The data were collected in real-world conditions, which enhances ecological validity and reflects the actual challenges faced by recreational athletes. Systematic tracking of training and regular measurements of BM and body composition allowed for precise monitoring of adaptations throughout the 26-week preparation period. Moreover, training load were evaluated using two complementary systems of intensity classification—one anchored to V_TM_ and the other to outcomes of a the Field-Based Tunning Test—thereby revealing differences in training structure that would otherwise remain undetected. Finally, the practical applicability of the described strategies, combined with the substantial improvements in performance achieved by both participants, provides valuable insights that may inspire athletes and coaches aiming for the sub-4-h marathon benchmark.

At the same time, this study is not without limitations. As a case study involving only two participants, the findings cannot be generalized to the broader population of recreational marathon runners. While such a design offers high ecological validity and detailed individual insights, it is inherently limited in its ability to establish causality or account for inter-individual variability. In addition, dietary intake was not directly monitored; instead, only recommendations were provided and body composition was assessed periodically. This lack of precise dietary records precludes verification of the hypothesis that the marked decline in muscle mass observed in Woman A was a consequence of low energy availability or insufficient protein intake. Moreover, no data were collected on broader lifestyle factors and on individual morphological and biomechanical characteristics, making it difficult to determine the exact mechanisms that led to the overuse injury sustained by Woman B. Additionally, the training intensity zones were not recalibrated throughout the 26-week period, which may have introduced minor inaccuracies in classifying exercise intensity over time. Similarly, the body mass and composition measurements obtained via bioelectrical impedance analyzer should be interpreted with caution, as the protocol did not account for potential measurement variability related to menstrual-cycle phase or hydration status. Furthermore, this report does not include a detailed participant perspective, which is recommended by the CARE guidelines to enhance the accuracy, transparency, and usefulness of case reports, nor does it include data allowing for the assessment of internal load (e.g., session rating of perceived exertion). Finally, it should be noted that the Field-Based Running Test used in this study was an original, non-validated protocol developed by the authors; however, it is considered by the authors to possess practical applicability and value in real-world training settings. These limitations should be taken into consideration when interpreting the results; however, they do not diminish the practical value of the presented data for athletes and coaches.

## Conclusion

This case report demonstrates that a multifactorial approach combining training load, nutritional education, body mass reduction of a rational magnitude, achieved primarily through fat mass loss, as well as optimizing the pacing strategy can lead to substantial improvements in marathon performance among eumenorrheic middle-age women aiming for a finishing time of around 4 h. Despite health issues in the final weeks of preparation, both participants achieved meaningful progress, illustrating the practical value of an integrated approach to marathon training. While the findings cannot be generalized, the insights provided here may inform coaches and athletes in designing effective preparation strategies for similar performance goals.

## Data Availability

The original contributions presented in the study are included in the article/supplementary material, further inquiries can be directed to the corresponding authors.
